# The Trajectory of Damaged-Base Eversion into the Active Site of Apurinic/Apyrimidinic Endonuclease APE1 Regulates This Enzyme’s Substrate Specificity

**DOI:** 10.3390/ijms252212287

**Published:** 2024-11-15

**Authors:** Anatoly A. Bulygin, Nikita A. Kuznetsov

**Affiliations:** 1 Institute of Chemical Biology and Fundamental Medicine, Siberian Branch of Russian Academy of Sciences, Novosibirsk 630090, Russia; skytolya@ya.ru; 2 Department of Natural Sciences, Novosibirsk State University, Novosibirsk 630090, Russia

**Keywords:** base excision repair, nucleotide incision repair, apurinic/apyrimidinic endonuclease, conformational dynamics, active-site plasticity, damaged nucleotide, nucleotide eversion

## Abstract

Apurinic/apyrimidinic endonuclease 1 (APE1) is responsible for the hydrolysis of the phosphodiester bond on the 5′ side of an apurinic/apyrimidinic site during base excision repair. Moreover, in DNA, this enzyme can recognize nucleotides containing such damaged bases as 5,6-dihydro-2′-deoxyuridine (DHU), 2′-deoxyuridine (dU), alpha-2′-deoxyadenosine (αA), and 1,*N*6-ethenoadenosine (εA). Previously, by pulsed electron–electron double resonance spectroscopy and pre-steady-state kinetic analysis, we have revealed multistep DNA rearrangements during the formation of the catalytic complex. In the present study, the modeling of the eversion trajectory of nucleotides with various damaged bases was performed by directed molecular dynamics simulations. It was found that each damaged base at the beginning of the eversion interacts with protein loop Val196-Arg201, which should be moved to enable further nucleotide eversion. This movement involves a shift in loop Val196-Arg201 away from loop Asn253-Thr257 and requires the disruption of contacts between these loops. The Glu260Ala substitution facilitates the separation of the two loops. Moreover, conformational changes in the Asn253-Thr257 loop should occur in the second half of the lesion eversion trajectory. All these perturbations within the protein globule tend to reduce steric interactions of each damaged base with the protein during the eversion of the nucleotide from DNA and movement to the active site. These perturbations are important determinants of substrate specificity of endonuclease APE1.

## 1. Introduction

Apurinic/apyrimidinic (AP) endonuclease APE1 is one of the enzymes participating in the DNA base excision repair pathway [1,2,3,4]. The main biological function of APE1 is thought to be the hydrolysis of the phosphodiester bond on the 5′ side of an AP site, resulting in 5′-deoxyribose phosphate and a 3′-OH group remaining at the ends of the strand break [5,6,7]. This is called AP endonuclease activity. In addition, several other minor functions have been discovered in APE1, one of which is the hydrolysis of the phosphodiester bond on the 5′ side of a nucleotide with some types of damaged bases, such as 5,6-dihydrouridine, the α-anomer of adenosine, and others [8,9,10,11]. Moreover, APE1 has 3′-phosphodiesterase, 3′-phosphatase [12,13], and 3′-5′-exonuclease [14,15,16,17] activities.

Many studies have addressed the mechanisms of substrate specificity of APE1 [18,19,20,21,22,23,24]. Our series of studies began with the modeling of complexes of human APE1 (and then of its homologs from *Danio rerio*, *Xenopus laevis*, and *Drosophila melanogaster*) with DNA duplexes containing various damaged nucleotides: an AP site, 5,6-dihydrouridine (DHU), α-adenosine (αA), or ethenoadenosine (εA) [25,26]. Our simulation results have revealed that one of the obstacles for a damaged nucleotide base during its eversion is a protein loop (at the boundary of the active-center pocket) containing amino acid residues (aa) 253 to 257 (the numbering is according to zebrafish APE1, zAPE1). This loop has steric conflicts with the bases; for a catalytically competent state to materialize, a change in the positioning of this loop is necessary toward an increase in the volume of the active-center pocket. It has been found [26] that residues Asn253 and Ala254 directly interact with the bases, and to shift the loop, it is necessary to disrupt contacts of residues Gly255 and Glu260 with surrounding structures (the numbering is according to zAPE1).

In the next work, we tested whether it is possible to alter the substrate specificity of zAPE1 by introducing substitutions Asn253Gly, Ala254Gly, and Glu260Ala [27]. It turned out that substituting Glu260Ala imparts a significant change in specificity to the enzyme: an enhancement of nucleotide incision repair (NIR) activity was observed on all NIR substrates tested, namely DHU, dU, αA, and εA. At the same time, the AP endonuclease activity of this mutant enzyme was several-fold lower.

These findings prompted us to gain a deeper understanding of the mechanisms underlying nucleotide eversion into the active center of AP endonucleases and to answer the question of why the greatest increase in the efficiency of the cleavage of NIR substrates is caused by the replacement of a residue that does not directly contact either a lesion or DNA as a whole. It is impossible to answer these questions via modeling or the analysis of any individual states of an enzyme–substrate complex. Therefore, it was decided to model the complete trajectories of nucleotide eversion into the active center of an APE1 enzyme.

Two enzymes were selected for the simulation here: the wild-type (WT) enzyme and zAPE1 mutant Glu260Ala. The emulation of the eversion process using the conventional molecular dynamics (MD) simulation was not feasible because the spontaneous occurrence of such large structural changes requires at least a long simulation period. Therefore, for this work, directed MD simulations were chosen, which consisted of applying artificial forces to atoms of the studied molecules, thereby facilitating the displacement of these atoms in a desired direction [28]. Thanks to the above approach, we were able to reveal the fundamental mechanism of nucleotide eversion into the active center of zAPE1 and find one of the possible reasons for the higher NIR activity when this enzyme carries the substituted Glu260Ala.

## 2. Results

### 2.1. Abasic Nucleotide Eversion

**Figure 1:**
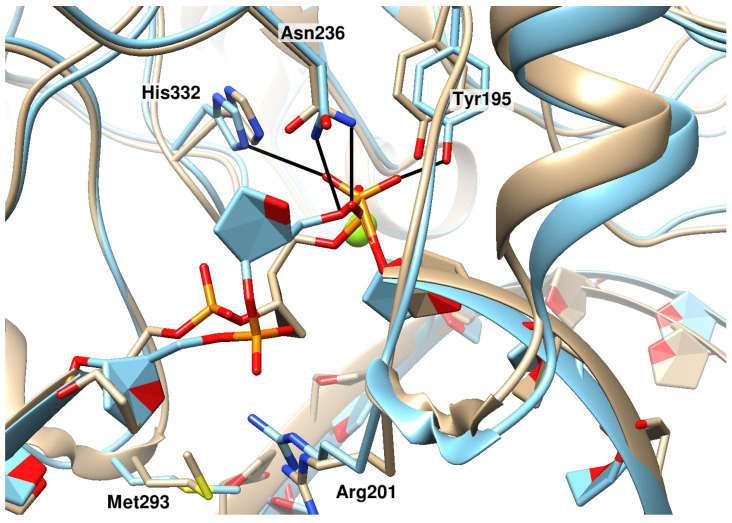
Initial (beige) and final (light blue) positionings of the F site in the trajectory of this lesion’s eversion into the active site of either WT zAPE1 or zAPE1 Glu260Ala. The colored lines denote the hydrogen bonds arising between the lesion and the enzyme.

The first to be modeled were complexes of enzymes with DNA duplexes containing an F site, a stable synthetic analog of an AP site. During the modeling of the F-site-containing DNA in a free state, the tetrahydrofuran residue stayed turned inside the DNA double helix quite stably. When this equilibrated DNA duplex was substituted into complexes involving either the WT or mutant enzyme (zAPE1), the tetrahydrofuran residue spontaneously got flipped out toward the interior of the active center of each enzyme within 1–2 ns, without encountering any obstacles, including those from protein structures. In the trajectory of the F-site eversion, only two relatively stable positionings could be distinguished: the initial one (inside the DNA helix) and the final positioning, namely a catalytically competent one (Figure 1). In the initial state, the phosphate group of the F site has only one hydrogen bond with the enzyme: between atoms O1P and Asn236 N^δ2^. In the final state, there are hydrogen bonds with all three residues of the active center.

### 2.2. Dihydrouridine Eversion

**Figure 2:**
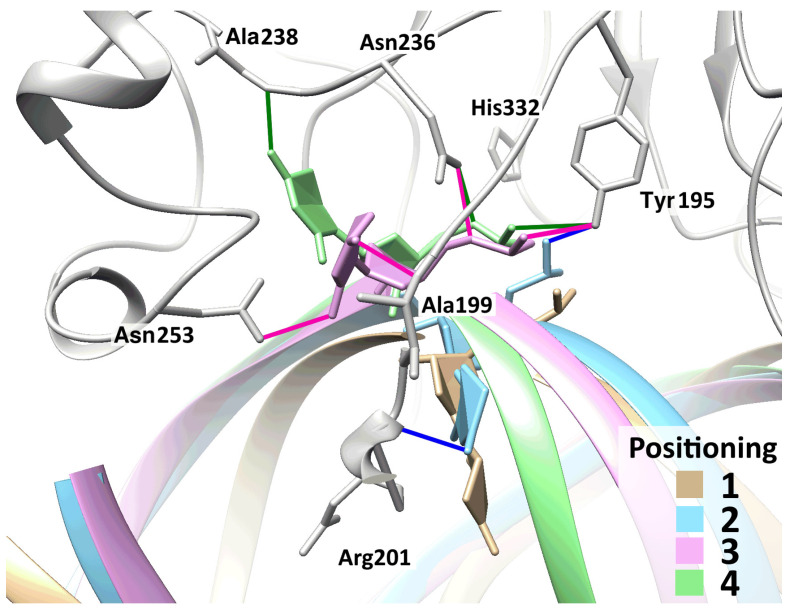
Representative structures characterizing the process of eversion of a damaged nucleotide containing DHU. In addition to the initial (beige) positioning and final (green) positioning, DHU undergoes two quasi-stable intermediate poses. In these positionings, the damaged base is engaged in specific contacts with residues of the DNA-binding site and active center of the enzyme; in the second positioning, DHU O4···Arg201 N (highlighted in blue); and in the third one, DHU O2···Asn253 N^δ2^ and DHU O4···Ala199 N (highlighted in pink).

When modeling the DNA duplex containing DHU, we obtained an expected result: the DHU base was not located inside the DNA helix owing to noncoplanarity. Dihydrouracil was found to be shifted toward the major groove of DNA and formed a single hydrogen bond with the opposite guanine: DHU O2···dG N1. In this positioning, it remained stable; only very brief severances of the hydrogen bond with guanine were observed. When the DNA duplex was substituted into the complex with the enzyme, and subsequent modeling was performed without the application of additional forces, the positioning of DHU did not change (Figure 2, beige structure), which meant that an additional force was needed.

The eversion of a nucleotide can be roughly represented as its rotation around the axis of the sugar–phosphate backbone. Therefore, atom N1 of pyrimidine bases and atom N9 of purine bases were chosen to serve as the ends of the levers on which the forces acted. The choice of these atoms allowed us to obtain a lever of the greatest length at which the orientation of the base was not affected during eversion. The second atoms in the pairs to which the force was applied were selected in accordance with two criteria. First, the direction of the force should approximately match the direction along which the nucleotide should presumably move. Second, for the greatest effectiveness of the force, the angle between the force vector and the N-glycosidic bond should be as close to 90° as possible. Owing to the second criterion, it was necessary to use two different atoms in pairs with N1/N9. This is because, among atoms of active-center residues, there were no atoms that were at a sufficient angle to the N-glycosidic bond in the initial state of the complex. Therefore, at the beginning of the modeling of the eversion trajectory, there was repulsion from the N-glycosidic nitrogen of the opposite base. Next, a force of attraction to the C atom of Asn236 was active, which is the closest atom to N-glycosidic nitrogens of the damaged nucleotide among atoms of the active center.

In complexes with either protein (WT or mutant), during the eversion of DHU, a one-time action of one additional force proved to be sufficient: at the beginning of the trajectory, a repulsive force between atoms of the opposite bases exerted its action (DHU N1 from dG N9), and then an attractive force between atoms DHU N1 and Asn236 C was active. The trajectory of DHU eversion contained, aside from the initial and final poses, two quasi-stable intermediates, which were also identical between the two enzymes (Figure 2). In the first (initial) positioning, DHU did not have hydrogen bonds with the enzyme, but during eversion, it gradually formed them (Figure 2). In the second positioning, the DHU O1P···Tyr195 OH bond emerged, and in the third, the DHU O5′···Asn236 N^δ2^ bond was observed as well. Additionally, other hydrogen bonds—specific to the intermediate poses—temporarily appeared, which are also depicted in Figure 2.

**Figure 3:**
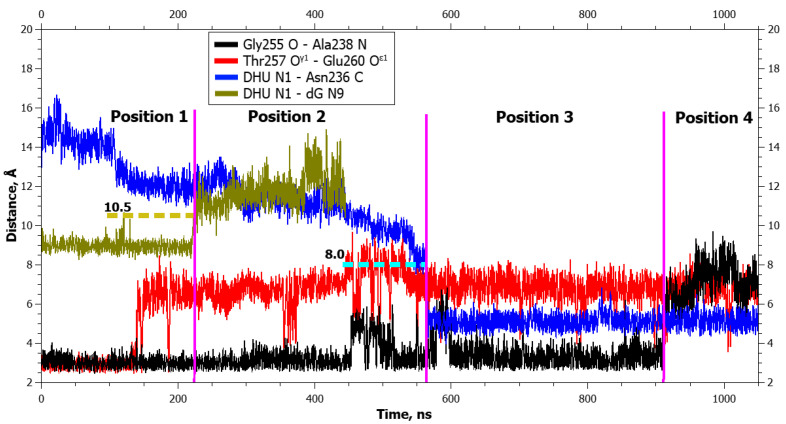
Dynamics of distances within pairs of atoms participating in hydrogen bonds in the Asn253-Thr257 loop (Gly255 O···Ala238 N and Thr257 O^γ^1···Glu260 O^ε^1) and between atoms involved in the application of additional forces (DHU N1···dG N9 and DHU N1···Asn236 C) during DHU eversion in the complex with the WT enzyme. The vertical lines indicate moments of transitions between states. The dotted lines denote the action of additional forces: the height means the distance up to which a force acted, whereas the left and right boundaries specify the time interval of the action.

**Figure 4:**
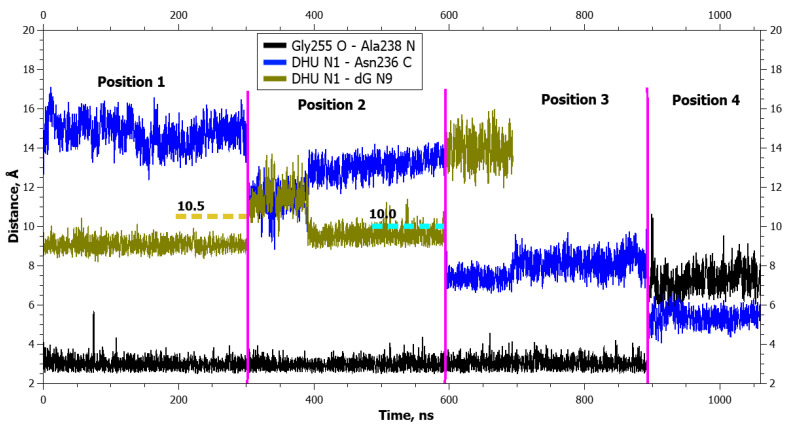
Dynamics of distances within pairs of atoms taking part in hydrogen bonds in loop Asn253-Thr257 (Gly255 O···Ala238 N) and between atoms involved in the application of additional forces (DHU N1···dG N9 and DHU N1···Asn236 C) during DHU eversion in the complex with the zAPE1 Glu260Ala enzyme. The vertical lines indicate moments of transitions between states. The dotted lines denote the action of additional forces: the height means the distance up to which a force acted, whereas the left and right boundaries specify the time interval of the action.

Aside from the gradual formation of bonds with the enzyme, distances between atoms of the damaged nucleotide and of amino acid residues of the active center decreased too, in an orderly manner. The graphs of dynamics of the distances (Figure 3 and Figure 4) also present the dynamics of distances between atoms DHU N1 and Asn236 C. It is evident from the graphs that the rather sharp jumps of average distances between the atoms took place during transitions between the poses. During the transition from the initial positioning to the second one (1 → 2), the distance between atoms DHU N1 and dG N9 (between which there was a repulsive force) increased first, but at the same time, the DHU N1...Asn236 C distance diminished too. During the second transition, the distance between DHU N1 and Asn236 C underwent a large spike. During the transition to the final positioning, no additional forces acted in any of the complexes (Table 1), and the damaged base spontaneously rotated into the active center with the concurrent rearrangement of the recognition loop and severance of the Gly255 O···Ala238 N bond. Furthermore, in mutant enzyme Glu260Ala at the end, some reduction in the DHU N1...Asn236 C distance was registered, which did not occur in the WT enzyme. It is worth noting that in the WT enzyme, hydrogen bond Thr257 O^γ1^···Glu260 O^ε2^ got severed already in the initial positioning under the action of the DHU N1···dG N9 force.

### 2.3. 2′-Deoxyuridine Eversion

**Figure 5:**
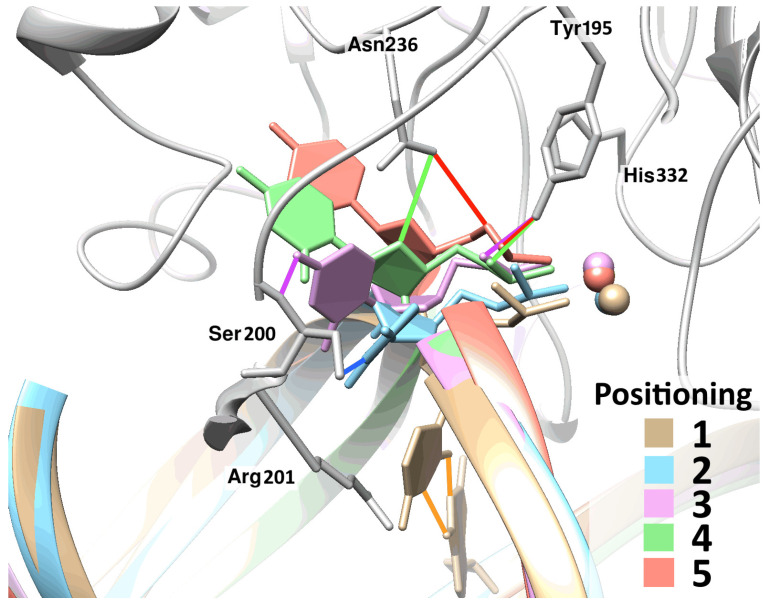
Representative structures characterizing the process of eversion of a damaged nucleotide containing dU. In addition to the initial (beige) and final (red) states, three quasi-stable intermediate positionings of the base during the eversion process are presented, with the second one shown in light blue, the third one in pink, and the fourth positioning in green. In these poses, the damaged base formed the following specific contacts with the enzyme: in the second positioning, dU N3···Ser200 O^γ^ (highlighted in blue), and in the third positioning, dU O4···Ser200 N (highlighted in green). In the fourth positioning, no specific uracil contact with the enzyme emerged.

Our modeling of a DNA duplex containing deoxyuridine (dU) revealed that uracil is located entirely within the DNA helix and is engaged in two concurrent hydrogen bonds with the opposite guanine (Figure 5, beige structure). When uracil got flipped out into the active center of the WT enzyme, it turned out that one additional force was not enough for the base to switch from the second positioning (Figure 5, light-blue structure) to the third one (Figure 5, pink structure). An obstacle in the path of the uracil was a protein loop containing aa Val196–Arg201, through which the base could not “pass” under the influence of any pulling force.

The second and third states of dU after eversion resembled the corresponding states of DHU. In the case of DHU, the base also moved past the Val196-Arg201 loop in the region of amino acid residue Ser200. The analogous transition of DHU proceeded, as already described, under the action of a single force of attraction to Asn236 C.

**Figure 6:**
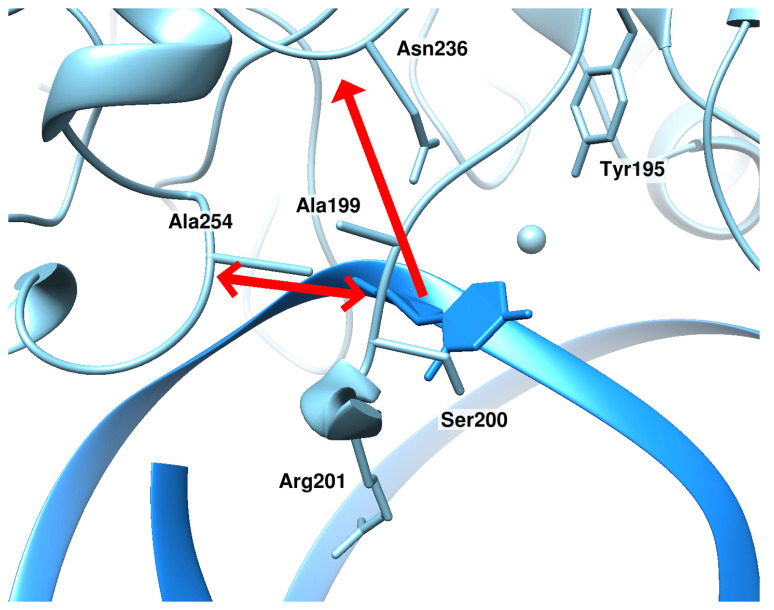
During eversion of dU, αA, and εA in certain positionings, two additional forces were applied simultaneously: a force attracting the base to the active site (the N-glycosidic atom of the nitrogenous base and Asn236 C: the vertical arrow) and a repulsive force between the two protein loops (atoms Ala254 C^α^ and Ser200 N: the horizontal double-headed arrow).

A comparison of the structure involving uracil in the second positioning with structures of all lesions in the final poses revealed that in the model of the intermediate complex with uracil, protein loops Val196-Arg201 and Asn253-Thr257 were positioned much closer to each other as compared to the other models. It was theorized that for further eversion of the base, it is necessary to extend the distance between these loops. This did not happen spontaneously during the modeling, and therefore it was decided to apply a second additional force: a repulsive force between the two protein loops (Figure 6). This force was applied to atoms Ala254 C^α^ and Ser200 N, which were located in the very center of the “mechanical” contact of loops Val196-Arg201 and Asn253-Gly255. The repulsive force acted until the distance between these atoms increased by at least 1 Å, while a minimal force was selected that was sufficient for repulsion to such a distance.

After the loops were moved apart by the additional force, the eversion of the base continued, and a transition to the third positioning occurred. In the subsequent two transitions to the final positioning, an additional repulsive force between the two protein loops was no longer required. In the last transition, the action of a base-pulling force was not required either.

During the modeling of dU eversion in the complex with zAPE1 mutant Glu260Ala, it was found that the uracil passed through the same quasi-stable intermediate states as in the complex with the WT enzyme; that is, the trajectories of dU eversion were the same between the two forms of the enzyme, as in the case of DHU. In the complex with zAPE1 mutant Glu260Ala, it was also necessary to apply a second additional force: a repulsive force between loops Val196-Arg201 and Asn253-Thr257. An important difference was that the magnitude of this force was noticeably smaller. The two protein loops in zAPE1 mutant Glu260Ala began to move away from each other already under the action of a force of 5 kJ/(mol·nm), in contrast to the WT enzyme, in which such a force had to be at least 7.5 kJ/(mol·nm); otherwise, the structural transformations did not take place (Table 2). It is noteworthy that, in both enzymes, after the transition to the third state, that is, after the base “passed through” protein loop Val196-Arg201, the action of additional forces was no longer required for further eversion.

**Figure 7:**
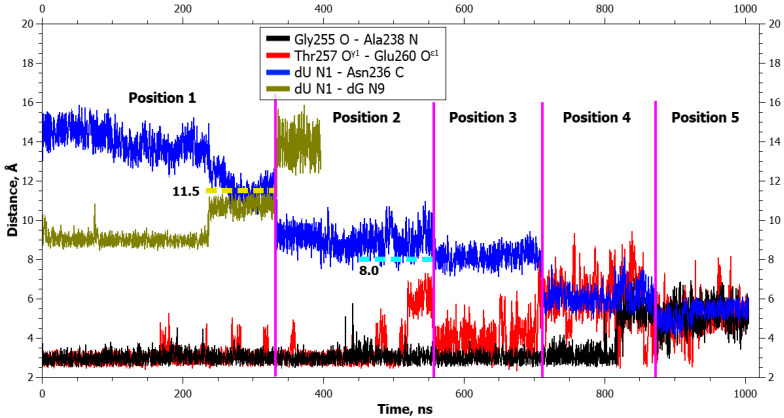
Dynamics of distances within pairs of atoms participating in hydrogen bonds in the Asn253-Thr257 loop (Gly255 O···Ala238 N and Thr257 O^γ1^···Glu260 O^ε1^) and between atoms involved in application of additional forces (dU N1···dG N9 and dU N1···Asn236 C) during dU eversion in the complex with WT zAPE1. The vertical lines indicate moments of transitions between states. The dotted lines denote the action of additional forces: the height means the distance up to which a force acted, whereas the left and right boundaries specify the time interval of the action.

**Figure 8:**
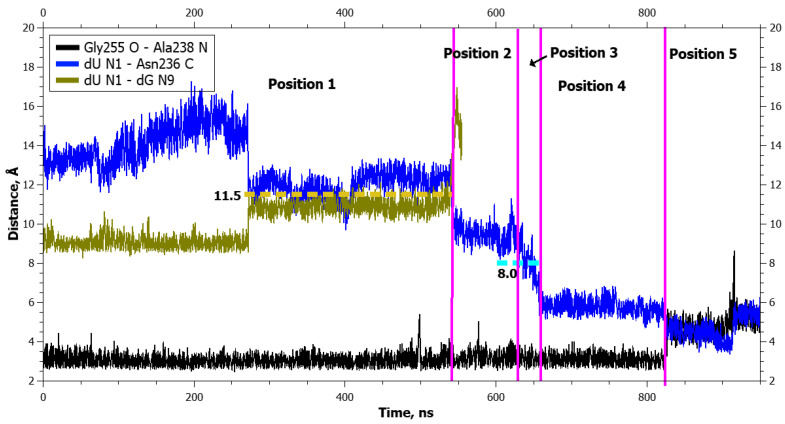
Dynamics of distances within pairs of atoms taking part in hydrogen bonds in loop Asn253-Thr257 (Gly255 O···Ala238 N) and between atoms involved in the application of additional forces (dU N1···dG N9 and dU N1···Asn236 C) during dU eversion in the complex with the zAPE1 Glu260Ala enzyme. The vertical lines indicate moments of transitions between states. The dotted lines denote the action of additional forces: the height means the distance up to which a force acted, whereas the left and right boundaries specify the time interval of the action.

In the graphs of the distance dynamics, it is obvious that (in contrast to the DHU case) when uracil was everted, hydrogen bond Thr257 O^γ1^···Glu260 O^ε2^ became destabilized in the WT enzyme, not at the very beginning but during the second transition between positionings (Figure 7). On the other hand, the hydrogen bond Gly255 O···Ala238 N (just as in the case of DHU) got severed during the transition to the final positioning in both enzymes (Figure 7 and Figure 8).

### 2.4. α-Adenosine Eversion

**Figure 9:**
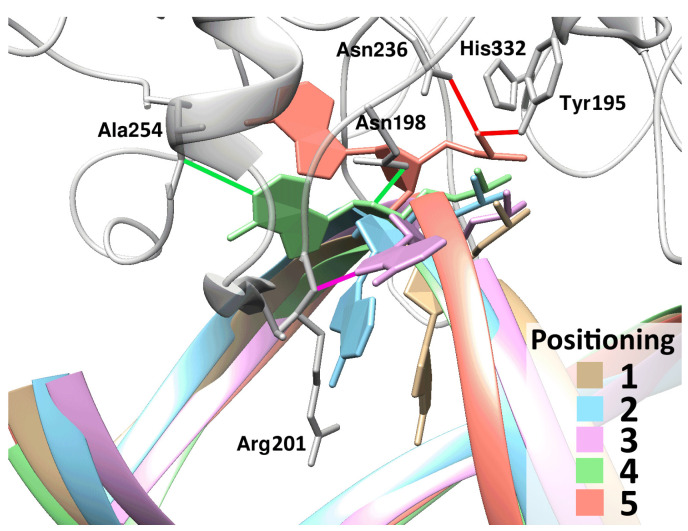
Representative structures characterizing the process of eversion of a damaged nucleotide containing αA. Three quasi-stable intermediate positionings of the nucleotide are displayed (light blue, pink, and green). In the third and fourth positionings, the base is engaged in the following specific contacts with the enzyme: in the third positioning, αA N7···Arg201 N (highlighted in pink), and in the fourth one, αA N1···Ala254 N and αA O4′···Asn198 N^δ2^ (highlighted in green). Bonds of the phosphate group with Asn236 N^δ2^ and Tyr195 O^η^ (highlighted in red) formed only in the final positioning.

In the model of the complex involving αA, the damaged nucleotide while being within DNA expectedly entered into two hydrogen bonds with the opposite thymine. The eversion of αA in both enzymes proceeded through three intermediate states (Figure 9). Just as in the case of uracil, in one of the transitions, namely in the transition to the fourth state, it was necessary to apply a repulsive force between the protein loops. Furthermore, it was found that the magnitudes of these forces were equal to those of the forces acting in the case of uracil (Table 3), and the mutant enzyme required a one-and-a-half times weaker force.

It should be pointed out that the final positioning of the αA base in the simulation of the full trajectory did not match the positioning in the models obtained in earlier studies [26]. In the first of such models [26], adenine proved to be turned with its five-membered ring toward the active-center pocket, whereas in our simulation of the eversion, this base, already in the last intermediate positioning, turned with its six-membered ring toward the active center and stayed in this pose until the end (Figure 9, green and red structures). Thus, the model of the αA-containing structure that was obtained earlier—in which the base was turned almost parallel to the DNA strand—can be interpreted as the result of an incomplete rearrangement of the lesion toward the optimal positioning, which was not completed due to insufficient simulation time.

**Figure 10:**
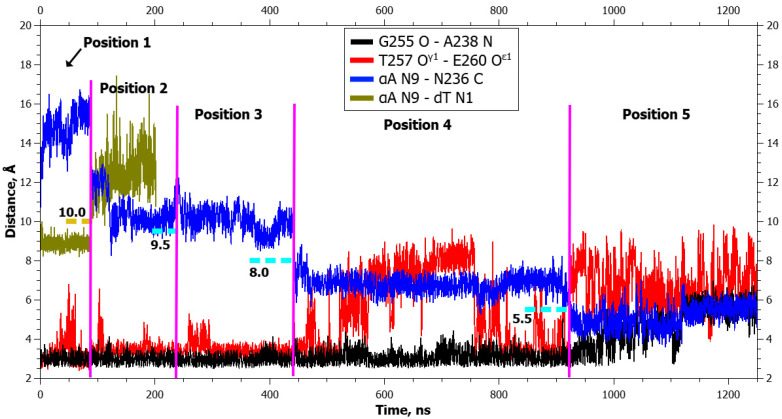
Dynamics of distances within pairs of atoms participating in hydrogen bonds in the Asn253-Thr257 loop (Gly255 O···Ala238 N and Thr257 O^γ1^···Glu260 O^ε1^) and between atoms involved in application of additional forces (αA N9···dT N1 and αA N9···Asn236 C) during αA eversion in the complex with the WT enzyme. The vertical lines indicate moments of transitions between states. The dotted lines denote the action of additional forces: the height means the distance up to which a force acted, whereas the left and right boundaries specify the time interval of the action.

**Figure 11:**
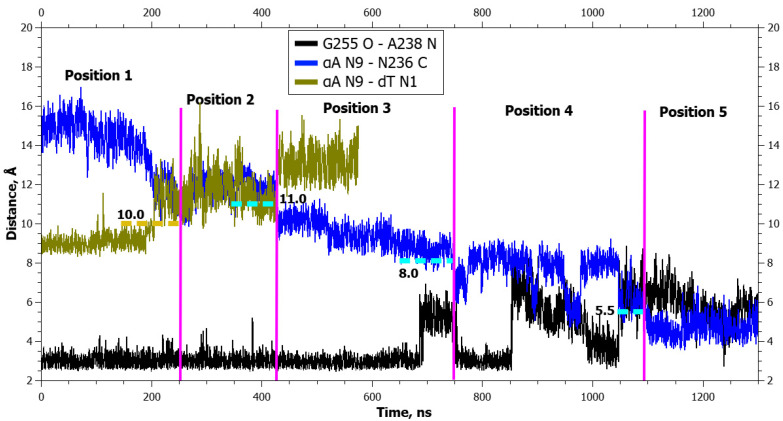
Dynamics of distances within pairs of atoms taking part in hydrogen bonds in loop Asn253-Thr257 (Gly255 O···Ala238 N) and between atoms involved in the application of additional forces (αA N9···dT N1 and αA N9···Asn236 C) during αA eversion in the complex with the zAPE1 Glu260Ala enzyme. The vertical lines indicate moments of transitions between states. The dotted lines denote the action of additional forces: the height means the distance up to which a force acted, whereas the left and right boundaries specify the time interval of the action.

In the WT enzyme, the Thr257 O^γ1^···Glu260 O^ε2^ bond lost stability in the fourth positioning (Figure 10). In the WT enzyme, the hydrogen bond Gly255 O···Ala238 N was severed only after the transition to the final positioning, whereas in the zAPE1 Glu260Ala enzyme, it became destabilized already during the transition to the fourth positioning (Figure 11).

### 2.5. ε-Adenosine Eversion

**Figure 12:**
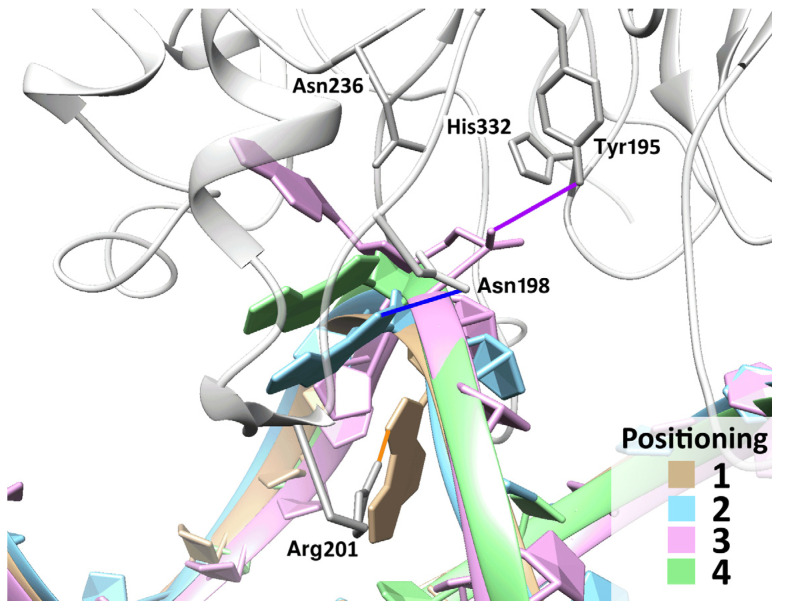
Representative structures characterizing the process of eversion of a damaged nucleotide containing εA. The base undergoes two quasi-stable intermediate positionings (highlighted in light blue and green). In the initial pose (beige), 1,N6-ethenoadenosine enters into a hydrogen bond with Arg201 via its N7 atom. In the second positioning, the εA N7···Asn198 N^δ2^ bond arises. The only bond (highlighted in purple) of the phosphate group with residues of the active center (Tyr195) formed only in the final positioning (pink).

The 1,N6-ethenoadenosine (εA) base in the initial positioning was found to be slightly shifted toward the major groove and did not form hydrogen bonds with the opposite base. During eversion, εA went through two intermediate states in the complexes with each form of the enzyme (Figure 12). Unexpectedly, it turned out that during the eversion of this nucleotide in both complexes, there was no need for an additional force pushing apart the protein loops on the path of the base (Table 4). εA was initially located on the correct side of protein loop Val196-Arg201 and apparently forced this loop to move away from loop Asn253-Thr257.

If we examine the structure of the complex involving εA in the initial positioning (Figure 12, beige structure), it becomes clear that εA has such a bulky base that it forces the side chain of Arg201—initially located opposite the damaged base—to shift to one side already during the modeling of the complex in the initial positioning. Moreover, the shift in all replicates is oriented toward the 5′ side of the damage. In cases of the other modeled lesions, the side chain of Arg201 either remained opposite the damage (in complexes with either dU or αA) or shifted to the 3′ side of the lesion (in complexes with DHU). Apparently, this behavior of Arg201 predetermines the direction of the entire trajectory.

**Figure 13:**
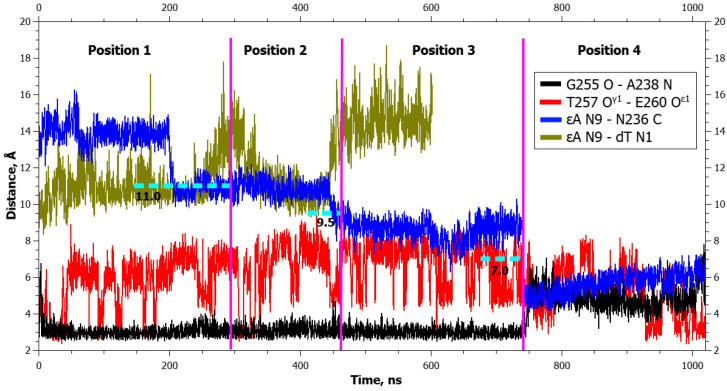
Dynamics of distances within pairs of atoms participating in hydrogen bonds in the Asn253-Thr257 loop (Gly255 O···Ala238 N and Thr257 O^γ1^···Glu260 O^ε1^) and between atoms involved in application of additional forces (εA N9···dT N1 and εA N9···Asn236 C) during εA eversion in the complex with WT zAPE1. The vertical lines indicate moments of transitions between states. The dotted lines denote the action of additional forces: the height means the distance up to which a force acted, whereas the left and right boundaries specify the time interval of the action.

**Figure 14:**
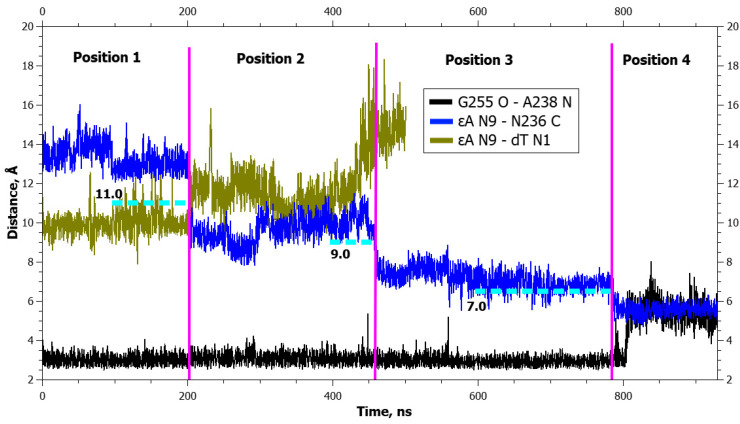
Dynamics of distances within pairs of atoms partaking in hydrogen bonds in loop Asn253-Thr257 (Gly255 O···Ala238 N) and between atoms involved in the application of additional forces (εA N9···dT N1 and εA N9···Asn236 C) during εA eversion in the complex with the zAPE1 Glu260Ala enzyme. The vertical lines indicate moments of transitions between states. The dotted lines denote the action of additional forces: the height means the distance up to which a force acted, whereas the left and right boundaries specify the time interval of the action.

In the WT enzyme, the Thr257 O^γ1^···Glu260 O^ε2^ bond became destabilized already in the initial positioning before any additional forces exerted their actions (Figure 13). In complexes with each form of the enzyme, the hydrogen bond Gly255 O···Ala238 N broke only after the transition to the final positioning (Figure 13 and Figure 14).

Another interesting observation is that, unlike αA, the εA base—though rotated around its N-glycosidic bond during the eversion—did not rotate by 180°. After the eversion, εA had the same positioning as in the final-state modeling in earlier papers [26,27].

## 3. Discussion

In previous articles, we have obtained important results suggesting that the “recognition” protein loop containing aa 253 to 257 plays an important role in the enzyme–substrate interaction [26]. Glutamate residue 260 immediately caught our attention because it largely determined the behavior of the recognition loop when the damaged base was located in the active center of the enzyme. Although it has been impossible to accurately determine the function of this residue during the modeling of only the final state, it was nevertheless decided later to construct mutants of zAPE1 to investigate the effect of aa substitutions in the recognition loop of zAPE1 Glu260Ala itself. Our second study confirmed the role of Glu260 in the enzyme’s specificity; the substitution of this residue with alanine significantly improved NIR activity while only slightly weakening the main AP endonuclease activity [27].

On the basis of these results, we hypothesized that the reasons for the change in specificity after substituting Glu260Ala can be found in the nucleotide eversion stage. Accordingly, in the current study, we examined in more detail and more deeply the interactions that occur between AP endonuclease zAPE1 and DNA containing various damaged nucleotides at the eversion stage. Our current technical and software capabilities do not permit us to realistically reproduce the nucleotide eversion process. Nevertheless, methods have been developed that make it possible to discern this process’s rough features, which can help to understand the fundamental mechanism. One such technique is directed MD simulations (used in this work).

**Figure 15:**
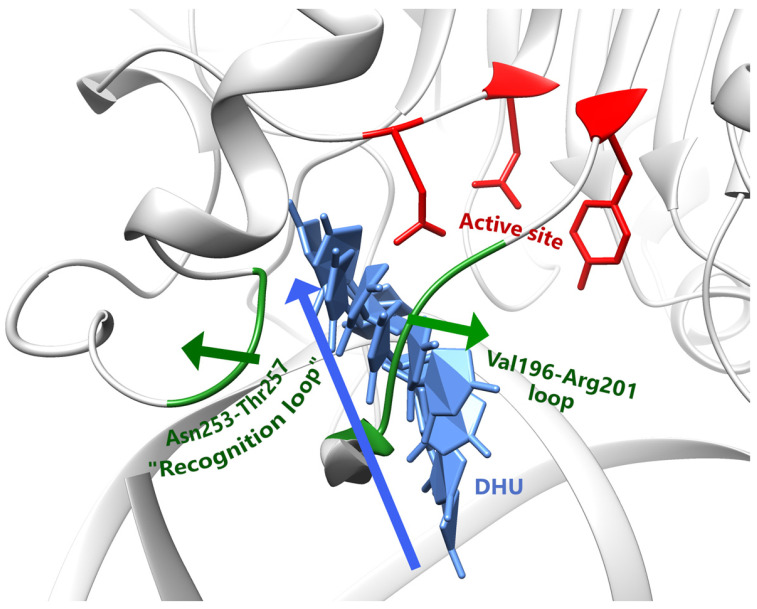
Overall view of the eversion trajectory in zAPE1 on the example of DHU. The blue arrow denotes nucleotide base movement direction, while the green arrows designate moving protein loops apart.

Via the modeling of the eversion trajectory of nucleotides with various damaged bases in directed MD simulations, it was found that the eversion of nucleotides occurs on the side of the DNA major groove (Figure 15). In this context, each damaged base from the very beginning of the eversion closely interacted with protein loop Val196-Arg201, which turned out to be the first obstacle on the path of the nucleotide. According to our simulation results, for successful eversion of a damaged nucleotide, this loop must change its positioning. This change involves a shift in loop Val196-Arg201 to one side from loop Asn253-Thr257, and this event in turn enables the nucleotide base to get between these loops and to continue the eversion. The two aforementioned loops interact with each other through several amino acid residues in each, but these processes are purely van der Waals interactions according to our structures. Nonetheless, after the substitution of Glu260Ala, distancing the two loops from each other is facilitated, which is most likely the reason for the improvement in NIR activity. A reason for the facilitated distancing of the loops is probably the elevated accessibility of the space between the loops for water molecules after substituting Glu260Ala, and this alteration in turn can disrupt other contacts.

In recognition loop Asn253-Thr257, conformational changes take place in the second half of the eversion trajectories, after the severance of the contacts of Glu260 with surrounding structures (if any); however, no differences were noted between the WT enzyme and zAPE1 mutant Glu260Ala. This means that amino acid residue 260 most likely no longer takes part in the conformational changes of the recognition loop.

## 4. Materials and Methods

The simulation was performed in GROMACS 2020.6 [29,30,31,32] by means of the AMBER ff99SB-ILDN force field [33,34] and BSC1 corrections [35]. Solvent molecules were represented by the TIP3P model [36]. Some water molecules were replaced by sodium ions to neutralize the charge of the system. The simulation was carried out with a system temperature of 300 K. The Verlet cutoff scheme [37] was chosen with a cutoff of 1.2 nm for both van der Waals and electrostatic interactions, whereas H bonds were constrained using the LINCS method [38]. Electrostatic interactions were computed in PME [39,40,41]. The energy of systems involving water was minimized first, and then, the two-stage equilibration of the system was carried out under the action of restraining forces acting on heavy atoms of the protein and DNA: 200 ps in the NVT ensemble and 200 ps in the NPT ensemble. After that, the main simulations were launched.

A model (obtained in our study [26]) of a complex of zAPE1 with an F site (a stable synthetic analog of an AP site) served as a basis for the preparatory stage of modeling. The DNA duplex was taken from it, and various damaged bases were introduced via substitution for the purpose of modeling in a free state. In this case, the damaged nucleotides were located inside the DNA helix. The structure of the protein from the same model was employed to create zAPE1 mutant Glu260Ala. Next, by means of WT zAPE1 and its mutant Glu260Ala, complexes with the previously obtained DNA duplex models were built. For these complexes, modeling was carried out without applying additional forces to balance the system.

In the case of directed MD simulations, we used the method of additional forces acting on certain atoms of DNA or protein in directions corresponding to expected conformational alterations. Three pairs of atoms were chosen to be subjected to additional forces:

1. The damaged base’s nitrogen atom, involved in the N-glycosidic bond, was repelled by the analogous nitrogen atom of the opposite base;
2. The damaged base’s nitrogen atom, involved in the N-glycosidic bond, was attracted to the Asn236 C atom;
3. The Ala254 C^α^ atom was repelled by the Ser200 N atom.

The additional forces used in Gromacs have a magnitude and a range of action (specified by means of a distance between atoms) that are chosen by the user. For the eversion of nucleotides, the force parameters were selected that were minimally sufficient for a transition between intermediate quasi-stable states within 200–300 ns. For each transition in each complex, MD trajectories were obtained in triplicate.

## 5. Conclusions

Thus, in this paper, it was demonstrated that an important role in the NIR specificity of APE1-like endonucleases is played by steric interactions of a damaged base with some protein structures during nucleotide flipping out from DNA and movement to the active center of the enzyme. If conformational flexibility is imparted to these protein structures, it weakens the steric conflicts, thereby enhancing NIR activity.

This work complements our series of papers devoted to studying the mechanisms of the zAPE1 specificity and the methods of its alteration. All the provided results, models, and predictions can be used for further research and development of APE1-based endonucleases that recognize various nucleotides with damaged bases.

## Figures and Tables

**Table 1:** Values of additional forces [kJ/(mol·nm)] in a complex with DHU-containing DNA, which is required for induced eversion of the nucleotide.

Transition	zAPE1	zAPE1 Glu260Ala	Force Type
1 → 2	8		Repulsion between atoms DHU N1 and dG N9
2 → 3	3		Attraction of atoms DHU N1 and Asn236 C
3 → 4	0		-

**Table 2:** Magnitudes [kJ/(mol·nm)] of additional forces in a complex involving dU that are required for induced eversion of this nucleotide. Attractive forces in the active center are identical, and only forces of repulsion of the protein loops differ.

Transition	zAPE1	zAPE1 Glu260Ala	Force Type
1 → 2	14		Repulsion of atoms dU N1 and dG N9
2 → 3	4		Attraction between atoms dU N1 and Asn236 C
	7.5	5	Repulsion of atoms Ala254 C^α^ and Ser200 N
3 → 4	0		-
4 → 5	0		-

**Table 3:** Values of additional forces [kJ/(mol·nm)] in a complex involving αA that are required for transitions of the nucleotide from one positioning to the next one during the eversion.

Transition	zAPE1	zAPE1 Glu260Ala	Force Type
1 → 2	10		Repulsion of atoms αA N9 and dG N9
2 → 3	1.5		Attraction between atoms αA N9 and Asn236 C
3 → 4	5	4	Attraction between atoms αA N9 and Asn236 C
	7.5	5	Repulsion of atoms Ala254 C^α^ and Ser200 N
4 → 5	6		Attraction between atoms αA N9 and Asn236 C

**Table 4:** Magnitudes [kJ/(mol·nm)] of additional forces in a complex involving εA that are required for transitions of the nucleotide from one positioning to the next one during eversion.

Transition	zAPE1	zAPE1 Glu260Ala	Force Type
1 → 2	7		Repulsion between atoms εA N9 and dG N9
2 → 3	4		Attraction of atoms εA N9 and Asn236 C
3 → 4	5		Attraction of atoms εA N9 and Asn236 C

## Data Availability

Data are available upon request to N.A.K. Tel. +7-(383)-363-5175, E-mail: nikita.kuznetsov@niboch.nsc.ru.
